# Postencephalitic amnesia with long term-working memory impairment: A case report

**DOI:** 10.1590/S1980-57642009DN20400022

**Published:** 2008

**Authors:** Beatriz Baldivia, Pablo Resende Saa, Maria Sheila Guimarães Rocha, Sonia Maria Dozzi Brucki

**Affiliations:** 1 Neuropsychologist. Service of Neurology from Santa Marcelina Hospital.; 2 Resident of Neurology.Service of Neurology from Santa Marcelina Hospital.; 3 Neurologist. Service of Neurology from Santa Marcelina Hospital.

**Keywords:** encephalitis, episodic memory impairment, naming deficit, confabulation

## Abstract

Herpes simplex virus encephalitis (HSVE) is an inflammation of the brain parenchyma caused by virus, leading to focal necrosis in medial temporal lobes, hippocampal complex and basal forebrain. Cognitively, HSVE is associated to many dysfunctions which vary according to the extent of the lesion. Episodic memory impairment is the most common sequelae following HSVE episodes, although others can occur. The aim of this case report was to describe the cognitive profile of a 42 year-old man who had extensive bilateral damage to the medial temporal lobe, insular bilateral and orbitofrontal cortices due to HSVE. Severe anterograde and retrograde amnesia, naming deficits, perseverative behaviors and confabulations were observed on neuropsychological assessment. We discussed the concept of long term-working memory based on this evaluation. These cognitive impairments corroborated HSVE previous findings in the literature.

## Abstract (pt)

Encefalite herpética (EH) é uma inflamação do parênquima cerebral causada pelo vírus herpes e associada à necrose focal nos lobos temporais mesiais, formação hipocampal e nas partes basais do córtex pré-frontal. Cognitivamente, a EH é relacionada a diversos prejuízos que variam de acordo com a extensão da lesão. Prejuízo na memória episódica é a seqüela mais freqüente, embora outros possam ocorrer. O objetivo deste relato de caso é descrever o perfil cognitivo de um homem de 42 anos que teve extensa lesão nos lobos temporais mesiais, nas ínsulas e nas regiões orbitofrontais em decorrência de um episódio de EH. Amnésias do tipo anterógrada e retrógrada, prejuízos na nomeação, comportamentos perseverativos e confabulações foram observadas pela avaliação neuropsicológica. Nós discutimos o conceito de memória operacional de longo prazo com vistas aos resultados obtidos. Os prejuízos cognitivos observados corroboram a literatura sobre EH.

Herpes simplex virus encephalitis (HSVE) affects mainly the medial and lateral temporal lobe and orbitofrontal cortices with bilateral or lateralized involvement.^1^ When damage occurs in the medial temporal lobe, deficits in episodic memory emerge, that is, the capacity to recall personally experienced and temporally specific events or episodes.^2^ Difficulties in encoding, storing and retrieving events learned from the past and related to new information may also occur, characterizing retrograde and anterograde amnesias, respectively. Damage to the temporal lobe in the dominant hemisphere is associated with semantic memory deficit and anomia, while lesions in the prefrontal cortex and subcortical areas which disrupt the interconnections within these areas, may produce executive dysfunction.^3^ Psychiatric symptoms, especially delusions and hallucinations, are not uncommon in HSVE.^4^

Due to the variety and extent of cognitive sequelae, the knowledge on the impact of HSVE on cognitive functioning is based on detailed single case reports. The aim of this study was to report the case of a patient who developed severe retrograde and anterograde amnesia, dysexecutive symptoms and impairment on language skills after HSVE.

## Method

### Case history

**Figure 1:**
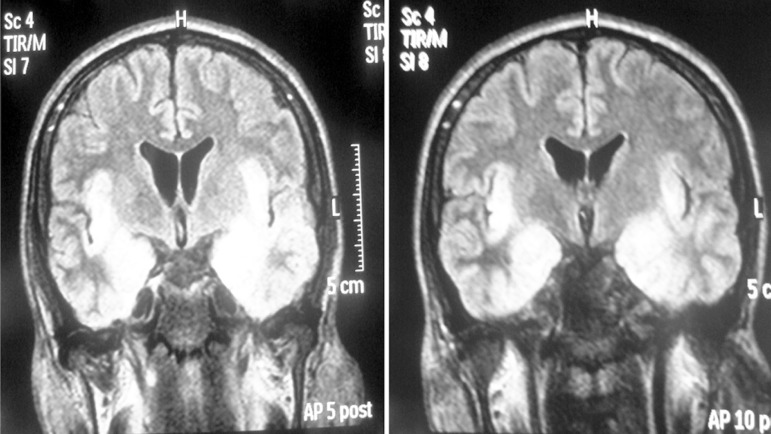
Hypersignal in flair acquisition on MRI in bilateral temporal lobes, extending to insula topography.

A 42-year-old, right handed man with 15 years of schooling, and previously employed as a pharmaceutics manager presented at our hospital after six days of fever and cough, a Glasgow Coma Scale of 14, and self-medicating with levofloxacin. He was hospitalized with diagnosis of pneumonia and delirium. After two days of hospital stay he was submitted to a lumbar puncture. CSF revealed 29 leukocytes (77% lymphocytes) and 131 red blood cells; protein of 84 mg/dl, and normal glucose level. Acyclovir (30 mg/kg/d) was administered after a diagnosis of herpetic encephalitis was reached, and subsequently confirmed by Magnetic Resonance Imaging exam (MRI) (Figure 1) and positive Polymerase Chain Reaction (PCR) for HSV1 in cerebrospinal fluid (CSF). He underwent 21 days of treatment. After two months, he returned for neuropsychological evaluation accompanied by his mother. At the first session, he claimed that he was present to accompany his mother during her consultation. Thus, he was unaware of his disabilities and his mother reported hyperorality, time and place disorientation and perseverative ideas related to his previous job. She also reported that he did not recognize some relatives and sometimes got her name wrong.

This case is reported following informed consent of the patient’s mother.

### Neuropsychological assessment

A formal neuropsychological assessment was carried out two months after the onset of symptoms. The test battery included screening tests (Mini Mental State Exam - MMSE),^5^ Frontal Assessment Battery^6^ (FAB) and Informant Questionnaire on Cognitive Decline in the Elderly (IQCODE)^7^ and outcomes scales: Disability Rating Scale^8^ (DRS) and episodic memory tests (Logical Memory and Visual Reproduction from WMS-R,^9^ RAVLT, Figure Complex of Rey), short-term and working memory tasks (Digit and Corsi forward and backward span), executive functions (Wisconsin Card Test, Stroop Color test, Trail Making part B, F A S verbal fluency) and language skills (Comprehension and Vocabulary from WAIS III,^10^ Boston naming test and verbal fluency: animals category). Due to the absence of normative data on the Brazilian population for some of these instruments adopted, the actual cognitive profile of V was compared to his previous level of cognitive functioning, considering the opinion of his mother and observing his behaviors regarding social skills, daily activities function and score on the IQCODE.

## Results

During the evaluation sessions, the patient was alert and his behavior was socially appropriate and cooperative. Nevertheless, he was disoriented in terms of time and place, had poor insight into his deficits and presented numerous confabulations. The main theme of the confabulations was that he had come into hospital because he was working as a pharmaceutical manager.

The scores on outcome scales, screening and neuropsychological tests are summarized in Table 1. Score on the Disability Rating Scale (DRS) revealed severe disability, with score of 7.5 points, distributed as follows: 1 for communication ability, 1 for toileting, 2.5 for level of functioning and 3 for employability. The Glasgow outcome scale also showed severe disability (score of 3). The IQCODE revealed that all functions were worse than ten years earlier, except for procedural skills. Performance on the MMSE confirmed time-place disorientation, impairment on 3-word delayed recall and difficulty in writing a phrase. The FAB showed impairment on conceptualization (similarities), reduced verbal fluency and mild disability in maintaining motor sequencing.

**Table 1:** Neuropsychological assessment results.

Cognitive function assessed	Tests	Raw score
Screening tests	MMSE	17
	FAB	10
Executive functions	Trail Making part B (time; errors)	265s; 1
	Stroop: card II (time; errors)	31s; 2
	Stroop: card III (time; errors)	34s; 0
	WSCT concepts	4
	WSCT errors	53
	WSCT perseverations	19
	RAVLT Σ A1-A5	19
	RAVLT proactive interference B	1
	RAVLT - immediate recall	0
Episodic memory	RAVLT - delayed recall	0
	RAVLT - recognition	8
	Memory logical - immediate recall	3
	Memory logical - delayed recall	0
	Visual reproduction: immediate recall	13
	Visual reproduction: delayed recall	0
	Figure complex of Rey: copy	36
	Figure complex of Rey: delayed recall	0
Language	Vocabulary	24
	Comprehension	13
	Fluency verbal : semantic category (animals)	6
	Fluency verbal : phonological category (F A S)	3/1/4
	Naming: Boston (spontaneous answers)	16
Short term memory	Digit Forward span	9
	Corsi Forward span	5
Working memory	Digit Backward span	8
	Corsi Backward span	6
Attention	Trail Making: part A (time; errors)	112s; 0
	Stroop: card I (time; errors)	40s; 0

MMSE, Mini Mental State Exam; FAB, Frontal Assessment Battery; WCST, Wisconsin Card Sorting Test; RAVLT, Rey Auditory Learning Test.

The more specific neuropsychological tests were selected based on the screening and scales results, and focused on the assessment of memory, executive functions and language skills. The patient presented severe anterograde amnesia (recall and recognition) for verbal and non-verbal materials. Retrograde amnesia was also observed from V’s discourse, since it was accentuated for personally experienced events and covered a period of 8 years (for example: V did not remember he had son, who is now 6 years’ old). It is important to mention that during free recall tasks V created false accounts, though related to an element picked up during the task, where this suggested confabulation induced by the memory test.

Despite the episodic memory deficits, performance on short-term memory tasks indicated preservation of this system and the independence of long and short term memories. In addition, V had good performance on working memory tests.

However, performance on language tests was impaired, revealing mild anomia, which was inconsistently facilitated by semantic cues (associated item), but not by phonological cues (first syllable). Performance on the Comprehension and Vocabulary test (a measure of verbal knowledge) was lower than average, with difficulty in proverb comprehension, and perseveration on the word ‘thing’ to explain the meaning of various expressions. On the FAS test, he produced fewer items than expected and committed a range of breakdown errors. He performed better on the semantic version of the task (animals category), but still scored outside the mean and had one breakdown error.

Assessment of executive functions revealed that V had impaired performance on the word fluency tests, conceptualization and motor sequencing tasks. On Trail Making part B, he committed one error (connected 11–12), suggesting a difficulty in rule retention.

## Discussion

This study described a patient with cognitive dysfunction in episodic memory, language skills and executive functioning after an episode of HSVE. The MRI exam showed that the patient had extensive bilateral damage to the medial temporal lobe, including hippocampus, left insular and orbitofrontal cortices.

Some case reports have suggested that dense and persistent retrograde amnesia may be caused by bilateral anterior lesions alone,^11-12^ while others suggest that a combination of damage in ventrolateral, prefrontal and temporopolar cortices might exacerbate memory impairment.^13^ Kapur et al.^11^ described the long-term magnetic resonance image (MRI) and neuropsychological profile in 10 patients who suffered from HSVE, and found that 60% of the cases had dense retrograde amnesia, whilst a smaller number of cases presented anterograde amnesia, naming and problem-solving difficulties. Our patient presented impairments in all of these cognitive functions, suggesting that the extension of the lesion had affected a larger number of cognitive functions. In addition, Kapur et al.^11^ found that bilateral damage to the hippocampal formation is closely correlated with the severity of amnesia. Akin to other amnesic patients,^14^ although severely impaired on episodic memory tests, V's short-term memory was intact as measured by digit span and working memory tasks. These results suggest a dissociate connection between the memory systems, usually found in amnesic patients such as HM, described by Scoville and Milner in 1957.^15^

On the other hand, these memory impairments could also be interpreted as a consequence of long-term working memory (LT-WM) deficit. Long-term working memory is defined as a retrieval schema in which information is encoded and stored in long-term memory, where it is then associated with its appropriate retrieval cues.^16^ When the information is selectively recalled, only the cue-based retrieval with an elaborate structure associating items from a given trial or context must be available in short-term memory (STM). In the case of patient V, he could not access the words previously acquired on the RAVLT test using the retrieval cue (recognition word list), suggesting a failure in both storage in long-term memory and cue-based retrieval.

These results conflict with findings on forward and backward digit span tasks, which showed V’s performance to be intact. This was analyzed in terms of the length of time the information was retained where the time which the information had to be maintained on the ST-WM for the digit span task was shorter than on the RAVLT, suggesting that longer tasks requiring a larger number of clusters impaired the retrieval of information in this patient’s LT-WM.

The performance of V on the Logical Memory task can also be attributed to a disability in LT-WM. When a story is heard, the content of this story form a structure that can be understood as a network of nodes or a cluster. Story comprehension requires information previously acquired to be retained and integrated with subsequent information, showing that the role of working memory in text comprehension is based only on transient activation of information to portions of structure in LTM as an extended working memory function. Patient V proved unable to retain and integrate the information, unlike the information retained to create other stories (confabulation).

Confabulations have been reported in association with HSVE outcome^17^ and associated with poor performance on executive tests.^18^ This may stem from amnesia overlaid with frontal dysexecutive impairment^19^ with a direct relationship between the degree of confabulation and degree of executive dysfunction. Moreover, cognitive deficit associated with confabulation is linked to specific executive dysfunctions, such as self-monitoring, set shifting and perseveration,^20^ and to tests tapping sustained attention and mental tracking, but has not been linked to concept formation, problem solving or verbal fluency.^21^ A recent study^22^ found a considerable variability between performance on tests of memory and executive functions in patients with focal frontal damage, but found striking evidence that the critical deficit for confabulation is anatomically located within the inferior medial frontal lobe, where orbital, medial and left lateral damage is related to personal episodic memory confabulations while right lateral damage confabulations are related to questions probing time orientation.

A strategic retrieval account of confabulation, indicated by failure in monitoring systems, associated with damage to ventromedial and orbitofrontal cortex is necessary for confabulation to occur.^23^ Moreover, deficits in other processes such as impaired memory, cue specification, temporal context confusion and content confusion may be necessary for a full confabulatory syndrome. These different components of strategic retrieval occurred in parallel in the case reported, showing that an extensive lesion caused by HSVE impaired the strategic retrieval processes, extending impairments to processes such as LT-WM that could aid the patient in remembering acquired information.

Impairments in naming ability, conceptualization and proverb comprehension have been ascribed to injury to the anterior lateral inferotemporal cortex, including the fusiform gyrus.^24^ According to Miceli et al.,^25^ when visual perceptual deficits have been ruled out, a failure in naming an object could stem from damage to the semantic system or the phonological output lexicon. When damage occurs to the semantic system, semantic errors would occur in parallel to deficits in comprehension.^26^ Based on V’s normal comprehension ability and the analysis of the types of breakdown errors presented on the semantic verbal fluency task, we suggest that damage to the phonological output lexicon underlies our patient’s naming deficits. This word retrieval deficit has also been previously described following an episode of viral meningoencephalitis.^27^

Verbal fluency depends on an efficient mechanism for searching the verbal knowledge store and is considered a frontal-executive task rather than a primary language function task. In this sense, the F A S task requires generation of a strategy for producing verbal output according to some rule or criterion nominated by the examiner. V committed diverse kinds of intrusions on this task, which were related to the phonological output lexicon (for example: he created a pseudo word that rhymed with a real word: he wished to say “apartamento”, but instead, said “adaptamento”), to episodic memory (repetitions), and executive functions (proper noun) dysfunctions.

Although HSVE patient performance on the WCST has been said to be unimpaired, poor performance on the word fluency test^27^ and deficits in executive functions such as abstraction, response inhibition or motor sequencing have commonly been found.^28^ This suggests that different lesion localizations within the frontal lobe is correlated with diverse impairments.

Orbitofrontal damage has been associated with disinhibition, inappropriate behaviors, personality changes, mood liability and distractibility.^29^ Moreover, damage to orbitofrontal and ventromedial portions of the frontal lobe have been associated with confabulation.^30^ Despite V’s appropriate behavior, symptoms of frontal syndrome were found in his perseverative behavior (i.e.: looking for his wallet many times during the sessions; stating that he was still working as a pharmaceutical manager) and in his spontaneous confabulations.

In sum, this case described the cognitive functioning of a young man who had suffered an extensive lesion to the bilateral temporal lobe which extended to the insula cortex. The neuropsychological assessment revealed the degree of cognitive impairment, while application of the outcome scales enabled the degree of deficits in functional level to be ascertained.
